# Beyond high‐throughput: leveraging plant phenotyping to improve understanding and prediction of plant growth through process‐based models

**DOI:** 10.1111/nph.71039

**Published:** 2026-03-02

**Authors:** To‐Chia Ting, D. Scott Mackay, Jinha Jung, Matthew P. Reynolds, Yang Yang, Diane R. Wang

**Affiliations:** ^1^ Department of Agronomy Purdue University West Lafayette IN 47907 USA; ^2^ Department of Geography University at Buffalo Buffalo NY 14226 USA; ^3^ School of Civil and Construction Engineering Purdue University West Lafayette IN 47907 USA; ^4^ International Maize and Wheat Improvement Center Texcoco 56237 Mexico; ^5^ Institute for Plant Sciences Purdue University West Lafayette IN 47907 USA

**Keywords:** carbon assimilation, image analysis, nitrogen uptake, physiological modeling, plant functional traits, spectral reflectance, water transport

## Abstract

The last decade has marked a period of rapid innovation in high‐throughput phenotyping (HTP) of plants. This includes the establishment of robotic phenotyping infrastructure, development of new sensors, and improvements in computation for downstream analysis. While HTP approaches have revolutionized data collection, meaningful insights into plant function require a yet deeper connection between resultant HTP‐based information and biological responses. We suggest that dynamic process‐based plant models, which simulate growth and physiology in a time‐explicit manner, can serve as a functional link between high‐throughput methods and whole‐plant mechanisms of growth. Using this framework, we review recent research that has leveraged HTP approaches for estimation of plant traits that are commonly used as process‐based model (PBM) variables. Through this analysis, we review successes and identify emerging directions for future research. Finally, we highlight the varied ways that HTP can be used in conjunction with PBMs as a tool to advance discovery and improve prediction of plant growth.

## Introduction

For the plant science community, the past decade has witnessed a wave of phenotyping innovation, driven largely by the development of sensors that quantify plant growth and physiology‐related metrics along with the improvement of robotic infrastructure which supports these sensors. The types of plant information that can be derived from these technologies are determined by the particular sensor modalities and their operating principles (Sun *et al*., 2022). For example, sensors detecting wavelengths shorter than 800 nm (e.g. X‐ray CT, red‐green‐blue (RGB), and Chl fluorescence) rely on atomic electron transitions and have been used to characterize various traits, such as root architecture (Tollner *et al*., 1987) from (Li *et al*., 2022), biomass (Tackenberg, 2007), or photosynthesis‐related measures, such as photosystem II efficiency (Baker, 2008), with strong absorption in red and far‐red regions. Solar‐induced fluorescence (SiF) has gained recent interest for outdoor photosynthesis assessment, although the temporal and spatial upscaling of SiF as proxies for photosynthetic traits remains to be resolved (Porcar‐Castell *et al*., 2014). Sensors detecting wavelengths in the region of 650 to 1550 nm or over 2500 nm, such as LiDAR and thermal, respectively, rely on molecular vibration transitions; LiDAR has been particularly useful for estimating structural canopy information, such as leaf area index (LAI) (Lin, 2015), while thermal sensors have been applied to understand energy balance, enabling inference of critical belowground traits, including root depth (Lopes & Reynolds, 2010) and capacity (Pinto & Reynolds, 2015). Spectral reflectance detects both atomic and molecular transitions in plant tissues. These spectral properties have been leveraged via vegetation indices to characterize changes in vegetation biomass (Rouse Jr. *et al*., 1974), plant architecture (e.g. LAI; Tucker, 1979), plant or vegetation water status (Peñuelas *et al*., 1993; Gao, 1996), and radiation use efficiency (RUE; Gamon *et al*., 1992; Garbulsky *et al*., 2011). Multispectral sensors were first adopted to achieve this task, and while they are still widely used, hyperspectral sensors now provide more comprehensive reflectance information, spanning 380–2500 nm (Curran, 1989; Doughty *et al*., 2011).

**Fig. 1:**
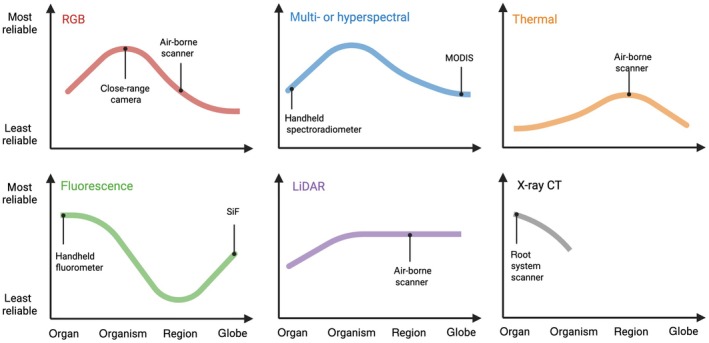
Reliability of different sensor modalities for proxying plant growth across scales. Traces indicate relative reliabilities (within each sensor modality) for red‐blue‐green (RGB), multi‐ or hyperspectral reflectance, thermal sensing, fluorescence, LiDAR, and X‐ray computed tomography. Sensors are not compared with each other, only within itself across scales of plant growth from organ to globe. Some examples of sensor implementation are annotated within each panel. This figure was created in BioRender (https://BioRender.com/nml9ext).

The different sensor modalities described above can proxy plant physiology and growth with varying levels of reliability across scales (Fig. 1) and have been deployed in both indoor and outdoor settings. Uncrewed aerial systems (UAS) and tractor‐based platforms are increasingly used for field‐based phenotyping (Shi *et al*., 2016), while various automated plant phenotyping facilities have been established around the world for controlled‐environment experiments, beginning *c*. 20 yr ago (Granier *et al*., 2006; e.g. The Plant Accelerator and High Resolution Plant Phenomics Centre (Australia); Ag Alumni Seed Phenotyping Facility and Nebraska Innovation Campus Greenhouse (USA); M3P, PhenoArch, PhenoDyn, and PhenoPsis (France); and Netherlands Plant Eco‐Phenotyping Center (the Netherlands)). This rich information stored in reflectance data has also had implications for larger spatial scale observations with the utilization of satellites. Low spatial resolution had been one drawback of traditional satellite data, but recent research has been able to remedy this by increasing spatial resolution to 50 cm (Tattaris *et al*., 2016; Pinto *et al*., 2023).

Despite these technological advancements, systematic connections between high‐throughput phenotyping (HTP) measurements with specific plant traits and processes that can mechanistically explain whole‐plant growth behavior, especially in response to abiotic stress, remain yet underdeveloped. To address this research gap, functional modeling has been identified as key to translating large amounts of phenotyping data into meaningful biological knowledge, for example, to derive basic mechanisms of complex processes, such as biomass accumulation (Tardieu *et al*., 2017). While there are different kinds of functional models, such as those that include explicit 3D representation, we focus our current synthesis of the phenotyping literature on studies relevant to dynamic physiological process‐based models (PBMs). These models can explicitly simulate complex interactions with feedback loops that occur over time and can incorporate multiple physiological mechanisms (Tardieu & Parent, 2017). PBMs, however, lack explicit 3D architectural representation and are therefore more computationally efficient than models that require detailed information on geometric properties. This makes them particularly useful for large‐scale studies, such as those that evaluate many genotypes or simulate multiple species at regional to global scales.

Here, our goal was to review the potential for linking HTP to PBMs to further mechanistic understanding of plant growth across scales. More systematic integration of these two approaches would help address key challenges in understanding how plant processes, such as carbon assimilation, water transport, and nutrient uptake and utilization, interact to affect organ‐level growth (e.g. leaf expansion, stem elongation, and root growth) and give rise to emergent canopy and rhizosphere response under variable conditions, which subsequently affect vegetation patterns at larger, for example global, scales. We (1) assemble plant traits that are commonly used as inputs or outputs of common PBMs; (2) synthesize recent research that has leveraged HTP approaches to estimate these traits, identifying successes and potential gaps; and finally, (3) examine effective examples of integrating HTP with PBM to develop guidance on future directions.

## What are process‐based models of plant growth? A primer

Dynamic (PBMs) of plant growth represent physiological functions (e.g. carbon assimilation, water transport, and nutrient uptake and utilization) as a series of mathematical equations. These governing equations are assembled into a single computer model driven by time‐varying environmental inputs (e.g. temperature, light, and precipitation) and parameterized by values that characterize species or genotype response to internal plant or exogenous environmental variables. Model outputs (fluxes and state variables) represent measurable plant traits that vary at each time step, which can influence behavior at the subsequent time step. Due to their time‐explicit nature, simulation output from PBMs can capture complex nonlinear behaviors as emergent properties. As such, PBMs have enabled investigation into the higher‐level growth consequences of manipulating lower‐level plant traits. Examples include examining biomass effects as a result of bioengineering C4 mechanisms into a C3 species (Yin & Struik, 2017); comparing the performance of cotton genotypes differing in stem hydraulic traits (Wang *et al*., 2020); simulating diverse root growth strategies *in silico* to provide evidence that trees under drought depend on established roots for water uptake vs growing roots *de novo* (Mackay *et al*., 2020); and prediction of biome boundaries using dynamic global vegetation models (DGVMs) and combinations of leaf and root functional traits (Fisher *et al*., 2015). However, the ability of PBMs to accurately simulate plant growth response hinges on extensive and diverse data for parameterization and evaluation (Box 1), underscoring the need for more high‐throughput approaches for phenotyping.

Box 1 **Data requirements for process‐based models**

Process‐based models (PBMs) require a wide array of data on plant growth and physiology for model parameterization and evaluation efforts. Parameterization is the process of assigning values to model inputs (excluding the environmental drivers), e.g. for *V*_cmax_ and *J*_max_. This can be done by gathering direct measurements (e.g. using a portable photosynthesis instrument to collect photosynthesis‐CO_2_ response curves and deriving *V*_cmax_ and *J*_max_), or through inverse modeling. Inverse modeling is used when there are observations of model outputs, such as biomass or leaf area index (LAI), often over multiple timepoints. Model input values are adjusted across a search space until differences between simulated outputs and observations are minimized. Once parameterized, PBMs are generally evaluated under different scenarios than those that were used for parameterization, for example, in a contrasting environment across multiple years, as in Bouidghaghen *et al*. (2023). This involves running the models with the same parameters but using different environmental drivers derived from those separate experiments. The modeled outputs are then compared with new observations to assess accuracy. Below: Conceptual overview of physiological PBMs showing inputs and outputs. *Environmental drivers* refer to time‐series of exogenous inputs, such as radiation, temperature, water input, and vapor pressure deficit. *Parameters* are single values that describe plant‐environment behavior (e.g. the response of stomatal conductance to vapor pressure deficit). Modeled outputs are shown to the right of the box and include *state variables* (e.g. LAI and biomass) and *fluxes* (e.g. carbon assimilation rate and transpiration rate).

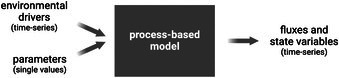

To provide the framework for this review, we first aggregated common variables used in plant PBMs by grouping physiological processes into three categories: carbon assimilation, water transport, and nutrient uptake and utilization. For each of these categories, four to six component models were selected, ranging from simple to more complex, and their input and output variables assembled (Supporting Information Fig. S1). The selection of these component models is described briefly below. Notably, representations of carbon, water, and nutrients are found in nearly all PBMs of plant growth whether they are developed to address questions in agriculture (e.g. crop growth models) or ecosystem research (e.g. earth system models; Table 1).

**Table 1:** Model variables.

Trait type	Model variable	Presence in agriculture process‐based models	Presence in ecosystem process‐based models
Functional (morphological)	Height	✓✓✓	✓
	Leaf angle	✓	✓✓
	Specific leaf area	✓	✓✓✓
	Tiller number	✓	✓
	Leaf area index^a^or leaf area	✓✓✓	✓✓✓
	Fraction of sunlit leaves	✓✓	✓✓✓
	Root traits^b^	✓	✓
Functional (phenological)^c^	Flowering time	✓✓✓	✓
	Leaf appearance rate	✓✓	✓
Functional (physiological)	Dark respiration	✓	✓
	Assimilation	✓✓	✓✓✓
	*V*_cmax_/*J*_max_	✓	✓✓✓
	Radiation use efficiency^a^	✓✓✓	✓
	Transpiration	✓	✓✓✓
	Stomatal conductance to water vapor	✓	✓✓✓
	Leaf water potential	✓	✓✓
	Leaf nitrogen	✓✓✓	✓✓✓
Performance (biomass)	Biomass	✓✓✓	✓✓✓

Only variables that had some support for estimation using high‐throughput approaches based on the current survey are shown here. Trait type refers to the trait framework of Violle *et al*. (2007).

^a^ Assuming a homogenous unit of land, this can be divided by plant number to compute value at the individual organism level.

^b^ Because high‐throughput phenotyping for root is not matured yet, traits like root density, root fraction, root radius, and root length are considered together.

^c^ Note that phenology is indeed represented in ecosystem process‐based models but not necessarily the two variables shown here. ✓ rarely or not present; ✓✓ sometimes present; ✓✓✓ frequently present.

For carbon assimilation, we considered a RUE model (Monteith, 1972) and the Farquhar, von Caemmerer and Berry C3 biochemical model (FvCB) of photosynthesis (Farquhar *et al*., 1980) along with canopy scaling options for the FvCB model (e.g. sunlit/shade (De Pury & Farquhar, 1997) vs multi‐layer (Goudriaan, 1977; Norman, 1982)). As plant PBMs often lack explicit representation of respiration processes, they are not included here. For water transport through the soil–plant‐atmosphere continuum, we identified sub‐models found in six larger models (WOFOST, APSIM, EPIC, DSSAT, SWAP, and CropSyst), which differ primarily in their degree of model abstraction (e.g. how they represent the driving force for water uptake) and are described and compared by Camargo & Kemanian (2016). For nutrient utilization, we focused on nitrogen and selected sub‐models from EPIC (Sharpley & Williams, 1990), CropSyst (Stöckle *et al*., 1994), CERES‐N (Godwin & Allan Jones, 1991), HERMES (Kersebaum, 1995, 2007). These models simulate the concentration of nitrogen in plant organs based on modeled demand and potential uptake.

The three major component processes described above ultimately function to influence the progression of plant growth across multiple levels, from organ to global scale. Growth and its associated traits can be viewed as a dynamic scaffold upon which other physiological functions interact and depend. For example, total leaf area underpins canopy transpiration, which impacts water uptake from soil. Despite the ubiquity of the term ‘growth’ in the plant sciences, its meaning is highly contextual and dependent upon spatiotemporal scale. These various perspectives on plant growth from cell to community level have been well‐articulated by Hilty *et al*. (2021), and for the current review, we selected growth traits (listed in parentheses) that represent each of the groups they outlined: mass (*biomass*), size (*height, leaf angle*), and indices (*tiller count, LAI, and specific leaf area* (*SLA*)). Due to their direct relevance for PBMs, we focused our scope to variables assayed at the individual plant level up to those measured on a land area basis. Notably, while these are measured at lower spatial scales, they are still relevant to models of plant growth at higher levels (e.g. regional and global scales) that are widely applied in ecological research, as these models use measurements taken on individual plants or over small land area. Because extracting belowground traits using high‐throughput means is relatively underdeveloped compared with aboveground traits, root characteristics were considered in a separate group. Finally, we included phenology, defined as the timing of life events driven by both biological and environmental factors, which influences all plant growth and physiology. For this, we surveyed *flowering time* (*FT*) and *leaf appearance rate* (*LAR*) as two phenological variables relevant to plant growth modeling, although recognizing there are many more.

**Fig. 2:**
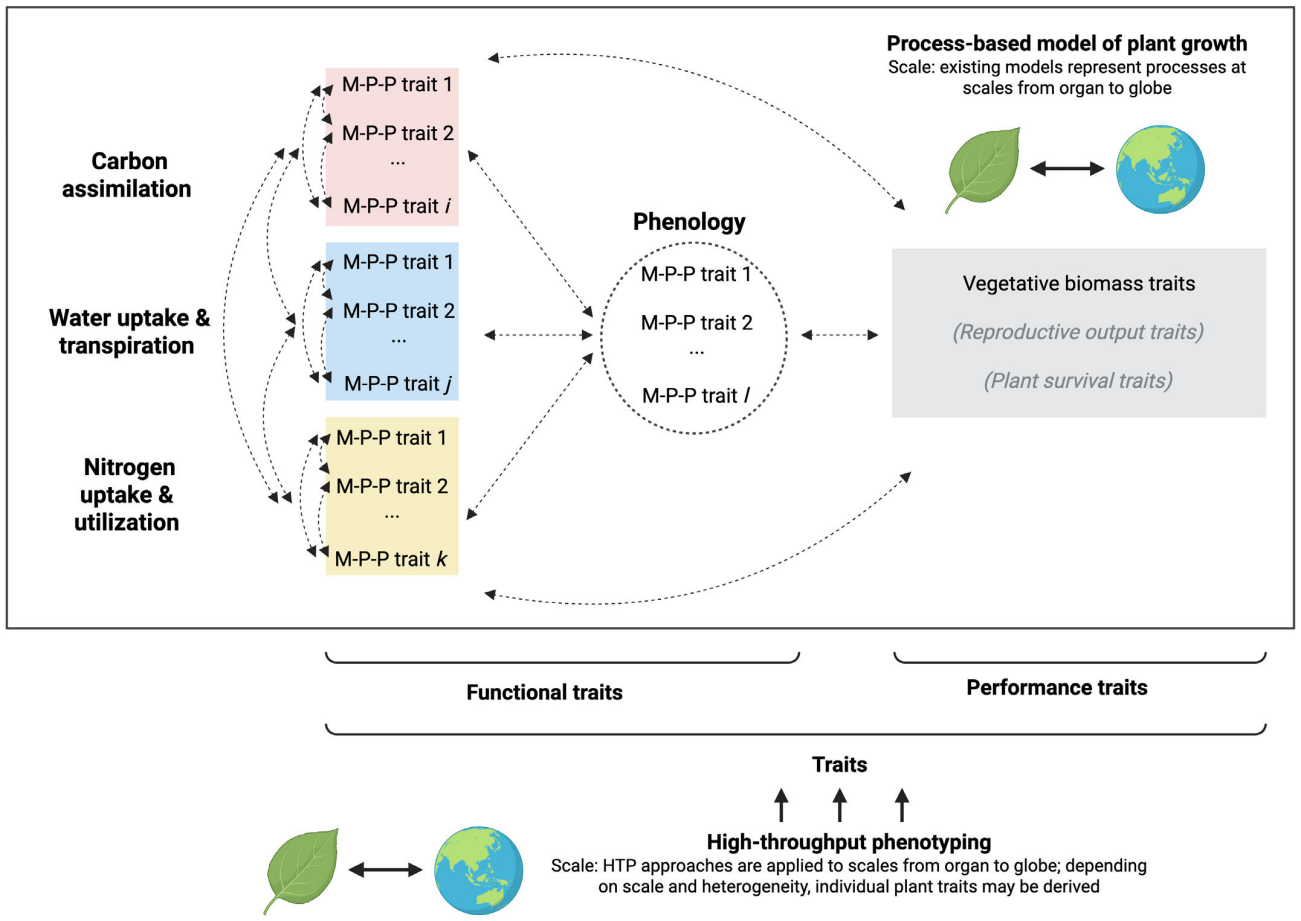
Representing process‐based model (PBM) variables as plant traits that can be estimated using high‐throughput phenotyping (HTP). PBMs simulate growth as an outcome of the interactions between carbon assimilation, water transport, nitrogen uptake and utilization, and phenology. Here, we map these components onto Violle *et al*.'s (2007) framework of plant traits, which categorizes traits into functional (i.e. morphology, physiology, and phenology) and performance traits (i.e. vegetative biomass, reproductive output, and plant survival). Growth metrics, per Hilty *et al*. (2021) include vegetative biomass (a performance trait) and morphology (the ‘M’ in M‐P‐P functional traits). Not shown is the feedback arrow between the ‘water uptake & transpiration’ component (blue box) and performance traits (gray box). Fig. S1 shows specific variables of these component models. This figure was created in BioRender (https://BioRender.com/mstbxuv).

To clarify the meaning of the term, ‘trait’, used throughout this paper, we follow the framework proposed by Violle *et al*. (2007), whereby traits are defined as standalone features (i.e. not requiring external references) measured at the level of individual, from cell to whole organism. Functional traits are morphological, physiological or phenological features that affect performance traits, which are measures of plant fitness. Performance traits include vegetative biomass, reproductive output, and plant survival. Here, we consider vegetative biomass as a plant performance trait that is relevant to existing PBMs of plant growth across scales. We avoid using the hard/soft distinction for traits, as this framing can be subjective (see Violle *et al*., 2007 for discussion). Figure 2; Table 1 present mappings of the PBM components discussed above onto Violle *et al*.'s trait framework. Combining this trait framework and the definitions of growth from Hilty *et al*. (2021), we note that growth metrics can be considered either functional traits (e.g. size metrics are morphological traits) or performance traits (e.g. mass metrics). HTP may be used to directly assay functional and performance traits at the scale of individual organism; HTP approaches implemented at higher spatial scales (e.g. on a land area basis) may also provide proxies of plant traits. These measures can be used to parameterize or evaluate PBMs at organism to global scale. Here, to evaluate the general utility of HTP approaches to estimate plant traits that serve as PBM variables, recent studies (2019–2024, with some exceptions) were systematically gathered from the Scopus database with full methods described in Methods S1.

## Which model variables may be evaluated with high‐throughput phenotyping?

HTP has rapidly expanded the scale with which PBM variables can be estimated across controlled and field conditions, but the ease and reliability of prediction differ substantially among traits. Overall, 19 of the PBM variables surveyed (Fig. S1) demonstrated recent progress for estimation using HTP approaches, including spectral reflectance, thermal, fluorescence, LiDAR, and X‐ray CT methods. Complete results are described in Notes S1; Table S1.

**Fig. 3:**
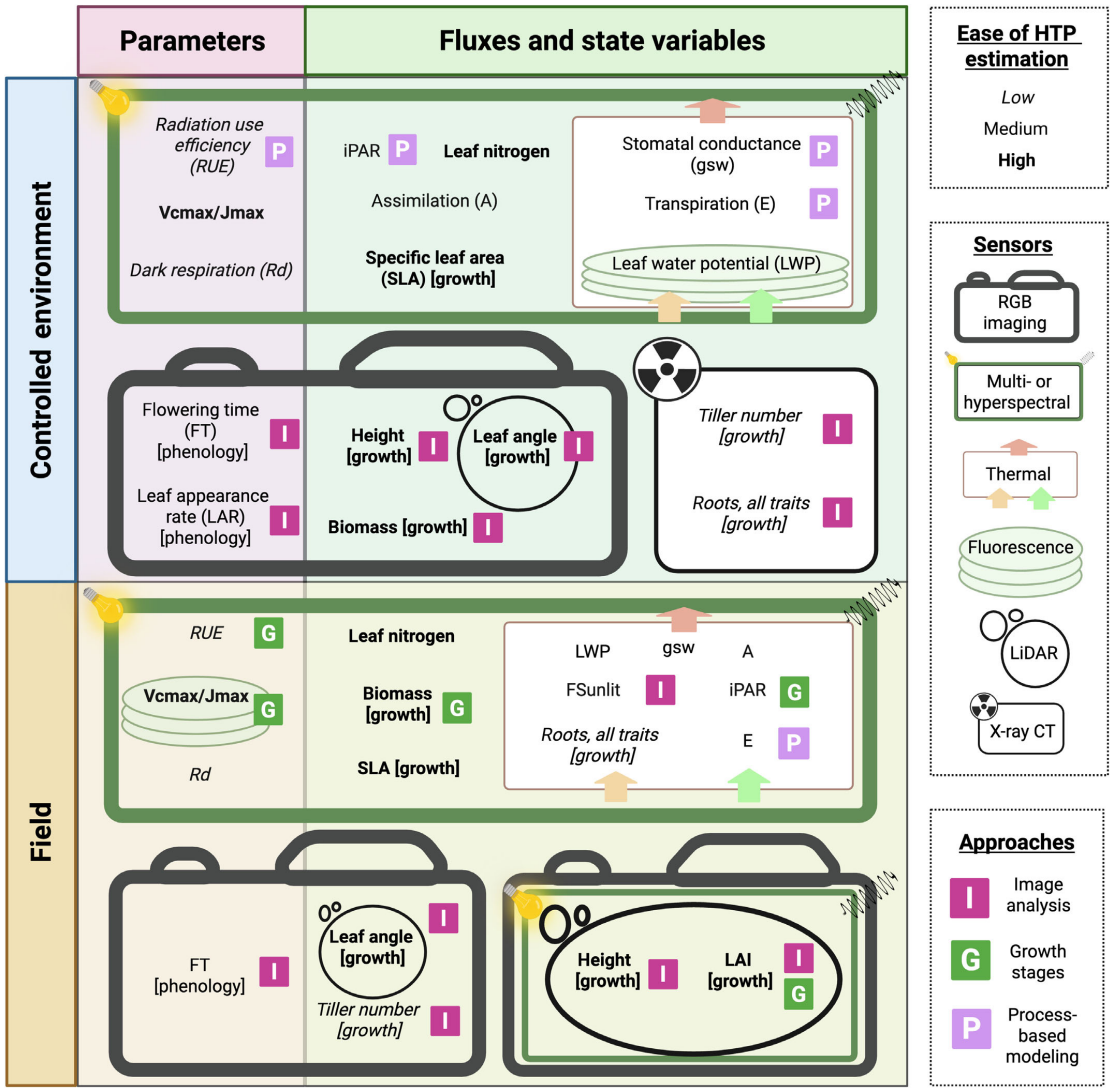
Current status of high‐throughput (HTP) approaches for estimating process‐based model variables. Model variables are shown here for their ease of estimation using high‐throughput approaches based on the current synthesis of the literature. Traits that can be estimated by more than one sensor are indicated by having one sensor icon embedded within the other one. Note that whether variables serve as inputs or outputs can vary depending on the model, but the most common use is presented here. For ‘ease of HTP estimation’ categories: low = use of HTP for prediction is rare or methods are underdeveloped; medium = some demonstrated success but may be inconsistent across experiments; high = HTP approaches are demonstrated across multiple species and settings. Estimation of some traits require additional approaches, such as image analysis for trait extraction (e.g. phenology related traits), growth‐stage‐dependent modeling or inversion of process‐based component models (e.g. for water transport traits). This figure was created in BioRender (https://BioRender.com/4rmocul).

Among the most predictable traits with spectral or structural signatures across environments and measurement platforms were those describing leaf biochemical capacity and canopy structure (Figs 3, S2; Table 2). Hyperspectral reflectance and SiF support reliable estimation of maximum carboxylation and electron‐transport rates (*V*_cmax_ and *J*_max_; Buchaillot *et al*., 2022; Li *et al*., 2020; Meacham‐Hensold *et al*., 2019; J. Wu *et al*., 2019), particularly when models explicitly account for growth stage or integrate phenology information (J. Wu *et al*., 2019; Li *et al*., 2020). The strength of these signals reflects well‐characterized optical relationships between chemical and physiological properties of plants, which suggests the potential for using HTP approaches to parameterize biochemical carbon assimilation models at scale (e.g. across large germplasm panels). In a similar vein, leaf nitrogen content is estimated with high confidence from hyperspectral data across a wide range of conditions (Coast *et al*., 2019; Ge *et al*., 2019; Zhi *et al*., 2022; Ting *et al*., 2023) leveraging its close functional linkage to Chl. However, prediction of nitrogen in other organs remains limited (Berger *et al*., 2020; Prey *et al*., 2020). For large experiments, the ability to estimate leaf nitrogen reduces destructive sampling and enables repeated measurements useful for model inversion or model evaluation.

**Table 2:** Summary of studies using high‐throughput methods to estimate input or output variables in process‐based models.

Traits	Settings	Sensors						Approaches			No. of species	No. of studies (range of sample size)	Range of trait values	Range of correlations
		RGB‐or RGB‐D	Multi‐or hyperspectral	Thermal	Fluorescence	LiDAR	X‐ray CT	Image analysis	Growth stages	PBM^a^				
Radiation use efficiency (RUE)	Field (g MJ^−1^)		*						*		2	2 (30–115)	0–4	0.61–0.83
	CE (g fresh weight PPFD mol^−1^ per plant)		*							*	1	1 (23)	7–11	0.84
iPAR	Field (MJ m^−2^)		*	*					*		1	2 (30)	200–1000	0.95
	CE (PPFD mol per plant)	*	*							*	1	1 (1600)^b^	0–30	NA
Dark respiration (Rd)	Both (μ mol m^−2^ s^−1^)		*								1	1 (1380)	0–1.4	0.73
Assimilation (A)	Both (μ mol m^−2^ s^−1^)		*								1	1 (98)	0–50	0.92
	Field (μ mol m^−2^ s^−1^)			*							1	1 (36)	0–20	0.85
	CE (μ mol m^−2^ s^−1^)		*								2	1 (147)	0–35	0.79
*V*_cmax_	Field (μ mol m^−2^ s^−1^)		*		*				*		24	4 (10–216)	0–359	0.46–0.94
	CE (μ mol m^−2^ s^−1^)		*								2	1 (158)	50–350	0.84
*J*_max_	Field (μ mol m^−2^ s^−1^)		*								2	2 (33–74)	150–700	0.77–0.87
	CE (μ mol m^−2^ s^−1^)		*								2	1 (158)	50–300	0.71
Fraction of sunlit leaves (FSunlit)	Field (unitless)		*	*				*			3	3 (1225)^c^	NA	0.90^d^
Transpiration (E)	Both (mmol m^−2^ s^−1^)		*								1	1 (98)	0–10	0.94
	Field (mm h^−1^)		*	*						*	1	1 (171)	0–0.8	0.71
	CE (mmol m^−2^ s^−1^)			*						*	3	2 (32)	0–5	0.81–0.89^e^
Stomatal conductance to water vapor (gsw)	Both (mol m^−2^ s^−1^)		*								1	1 (98)	0–0.6	0.79
	Field (mol m^−2^ s^−1^)			*							1	1 (30)	0–0.35	0.86
	CE (mol m^−2^ s^−1^)			*						*	5	2 (< 50)	0–0.7	0.73–0.98
Leaf water potential (LWP)	Both (‐MPa)		*								1	1 (98)	0.4–2	0.63
	Field (‐MPa)			*							1	1 (27)	0–3	0.85
	CE (‐MPa)			*	*					*	4	1 (28)	0–12.5	0.9
Leaf nitrogen (LeafN)	Both (%)		*								2	2 (838–1380)	0–8	0.92–0.95
	Field (%, g m^−2^)		*								653	3 (129–651)	0.07–0.39^f^	0.49‐0.9
	CE (%)		*								1	1 (88)	0–4	0.89
Flowering time (FT)	Field (h)		*								1	1 (720)	9–14	0.70
	CE (date)	*									1	1 (104)	20–40	0.88
Leaf appearance rate (LAR)	CE (leaf stage)	*									1	1 (2289)	1–14	0.97
Height	Field (cm)	*	*			*		*			4	4 (120–264)	0–250	0.96–0.97
	CE (cm)	*						*			2	1 (100–369)	0–200	0.81–0.99
Leaf angle	Field (degree)	*				*		*			1	2 (*c*. 170)^b^	0–90	0.91
	CE (degree)	*				*		*			3	3 (100–1600)^b^	0–100	0.84
Biomass	Field (g m^−2^)		*						*		2	3 (30–247)	0–3000	0.6–0.91
	CE (g per plant)	*						*			2	2 (25–54)	0–300	0.95–0.99
Leaf area index (LAI)	Field (m^2^ m^−2^)	*	*			*		*	*		4	5 (11‐96)^b^	5–30	0.82‐0.93
Specific leaf area (SLA)	Both (cm^2^ g^−1^)		*								2	3 (98–1414)	100–704	0.75–0.88
	Field (cm^2^ g^−1^)		*								652	2 (137–169)	34–333	0.82–0.96
Tiller number	Field (m^−1^)	*						*			1	1 (36)	0–300	0.93
	CE (plant^−1^)						*				1	1 (35)	0–25	0.93
Root dry weight/root traits	Field (g m^−2^)			*							1	1 (36)	0–70	0.94
	CE						*	*			3	2	NA	NA

Studies are grouped into experimental settings (field or controlled environment) with the corresponding units. Sensors and approaches used are reported along with the no. of species, no. of studies, range of trait values and range of correlations used to evaluate these methods. Asterisk indicates sensors and approaches applied to estimate each trait. CE, controlled‐environment; iPAR, intercepted photosynthetically active radiation; *J*_max_, maximum rate of electron transport; LiDAR, light detection and ranging; PBM, process‐based model; RGB, red‐green‐blue; RGB‐D, red‐green‐blue‐depth; *V*_cmax_, maximum rate of RuBisCO‐catalyzed carboxylation; X‐ray CT, X‐ray computed tomography.

^a^ When process‐based models are used, leaf area or biomass, estimated from RGB images, are typical inputs, as in the case of predicting RUE and iPAR.

^b^ One of the studies proposed an analysis pipeline, but no model metrics reported (i.e. no ground‐reference comparison).

^c^ The value was from one study only. The other two studies did not have ground‐reference comparisons.

^d^ The metric reported was classification accuracy.

^e^ One of the studies compared transpiration estimated from the Jarvis–Stewart model.

^f^ Only studies with unit of (g m^−2^) had information on range of traits.

Certain growth‐related traits are also estimated well using high‐throughput approaches. Plant *height* is routinely and accurately derived from RGB image analysis pipelines (Gehan *et al*., 2017), while *leaf angle* and *LAI*, key parameters for scaling leaf to canopy photosynthesis, can be captured through 3D reconstruction from RGB, multispectral, or LiDAR point clouds (Che *et al*., 2020; Liu *et al*., 2021; Sabag *et al*., 2024; Zhang *et al*., 2024; Shi *et al*., 2025). *Aboveground biomass*, a performance trait, shows moderate‐to‐strong predictability using HTP information collected from multiple sensors (Robles‐Zazueta *et al*., 2021; Pokhrel *et al*., 2023). Finally, *SLA*, central to the leaf economic spectrum (Wright *et al*., 2004), demonstrates high–moderate to high correlations with hyperspectral reflectance across crops and natural vegetation (Ali *et al*., 2017; Coast *et al*., 2019; Ge *et al*., 2019; Cotrozzi *et al*., 2020; Zhi *et al*., 2022), further underscoring the coupling between spectral signatures and leaf chemical properties. Taken together, these results highlight *V*_cmax_, *J*_max_, and *leaf nitrogen*, along with *height, leaf angle, LAI*, *biomass*, and *SLA* as a core set of PBM variables that are reliably predicted using HTP approaches, enabling application of models at scale.

**Fig. 4:**
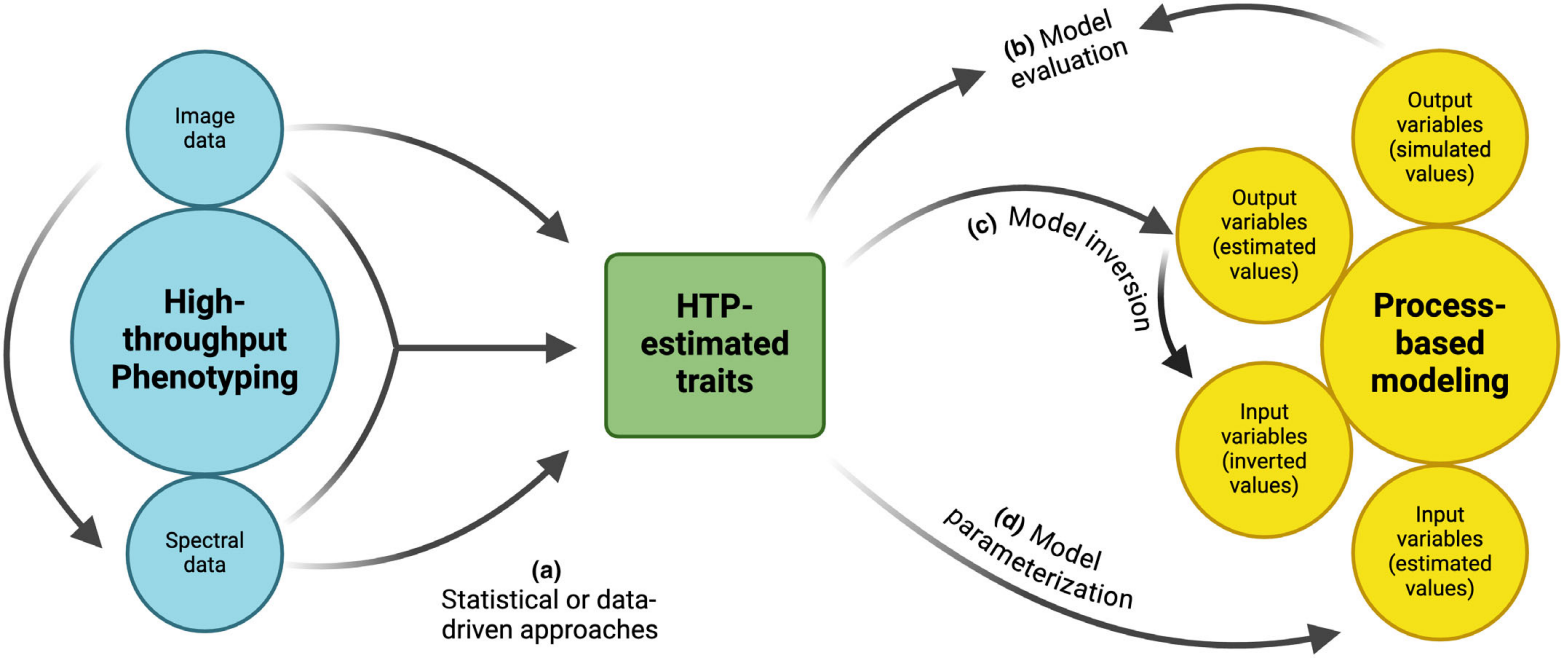
Opportunities to link high‐throughput phenotyping (HTP) with process‐based model (PBM) of plant growth. (a) High‐throughput plant phenotyping approaches yield image and/or spectral information, which may be used alone or together for estimation of plant functional traits that are associated with PBM inputs and outputs. This estimation is carried out using statistical or data‐driven approaches, such as predicting leaf nitrogen with partial least squares regression using hyperspectral reflectance data. Once functional traits are estimated, they can be linked to PBMs in a variety of ways. (b) Time‐series HTP‐derived traits can be compared with simulated state variables (e.g. LAI) for model evaluation. (c) Alternatively, they can be used on their own for model inversion to derive low‐throughput traits; for example, estimating maximum stomatal conductance by inverting a transpiration sub‐model from HTP‐derived transpiration and plant architecture. (d) HTP‐estimated traits can also be used directly as inputs for model parameterization, such as in the case of *V*_cmax_ and *J*_max_. This figure was created in BioRender (https://BioRender.com/fdc2qmh).

By contrast, some PBM variables demonstrate very limited or inconsistent predictability using HTP approaches. From survey results, we observe that estimation of *dark respiration* (Rd) with spectral information is rarely attempted, likely because ground‐reference nighttime measurements themselves are logistically challenging to collect; however, the single recent study we did find reported moderate success in making predictions using hyperspectral reflectance (Coast *et al*., 2019). Similarly, while RUE could be estimated using hyperspectral reflectance with moderate accuracy, prediction depends strongly on growth stage (Robles‐Zazueta *et al*., 2021) and growth habit (e.g. determinate or indeterminate; Pokhrel *et al*., 2023), limiting generalizability. These results suggest that *Rd* and *RUE* may be parameters best suited to estimate using model inversion rather than using HTP approaches for direct parameterization (Fig. 4).

Architectural traits where physical access or organ occlusion affect prediction represent potential areas for future HTP improvement. Belowground root traits currently pose the greatest obvious challenges. While transparent media and X‐ray CT enable nondestructive imaging of root length, radius, and density, extension of these methods across substrates and environments remains rather limited; field proxies such as canopy temperature can differentiate aspects of root performance under stress and are useful for models that simulate energy balance; however, root architecture evaluation itself cannot be explicitly addressed (Lopes & Reynolds, 2010; Pinto & Reynolds, 2015; Herrero‐Huerta *et al*., 2021; Zhu *et al*., 2022). Finally, for aboveground growth traits such as *tiller number*, self‐occlusion currently limits its prediction to early growth stages (Roth *et al*., 2020; D. Wu *et al*., 2019). Recent development of 3D plant reconstruction methods using novel ‘weakly supervised deep learning’ (i.e. vs more labor intensive standard supervised deep learning) are able to segment previously occluded plant organs (Luo *et al*., 2023; Saeed *et al*., 2023); advancements in this area may provide solutions for estimating currently inaccessible aboveground traits in the future.

The remaining eight traits demonstrate moderate predictability, where using HTP approaches for estimation have not been widely adopted (Figs 3, S2). These variables include gas exchange, such as *net assimilation* (*A*) (Cotrozzi *et al*., 2020; Buchaillot *et al*., 2022; Ramírez‐Cuesta *et al*., 2022), *stomatal conductance* (*gsw*) and *transpiration* (*E*) (Beverly *et al*., 2020; Cotrozzi *et al*., 2020; Ramírez‐Cuesta *et al*., 2022), predicted from hyperspectral features or thermal measurements. At plot scale, transpiration prediction improves when thermal imagery is integrated with energy‐balance models (Gómez‐Candón *et al*., 2021); at whole‐plant scale, high temporal frequency weight change from load‐cells coupled with RGB‐derived leaf area provides informative dynamics to invert biophysical models to plant transpiration (Eyland *et al*., 2022) and whole‐plant conductance (Prado *et al*., 2018). Similarly, water status metrics such as *leaf water potential* (*LWP*) are estimable with hyperspectral data but typically strengthen when thermal or fluorescence signals are included because changes in water status alter energy balance (Beverly *et al*., 2020; Cotrozzi *et al*., 2020; Ramírez‐Cuesta *et al*., 2022).

Intercepted photosynthetically active radiation, *iPAR*, is needed for RUE models and is moderately predicted from vegetation indices and canopy temperature when growth stage is accounted for Pokhrel *et al*. (2023); Robles‐Zazueta *et al*. (2021). Complementary approaches that combine RGB‐based biomass with measured light environments reveal seasonal dynamics in *RUE*, highlighting the value of multi‐sensor experimental designs (Cabrera‐Bosquet *et al*., 2016). Similarly, another canopy‐related variable, fraction of sunlit leaves (*FSunlit*), can be predicted from radiance data from hyperspectral images or using thermal imagery, particularly when machine learning is used to classify pixels (Meacham‐Hensold *et al*., 2020; Pantelidakis *et al*., 2022; Ramírez‐Cuesta *et al*., 2022). Finally, phenology traits such as FT and *LAR* have demonstrated moderate predictability through image analysis. For FT, RGB, multispectral and NIR‐green‐blue sensors have been used with success across large intraspecific panels (Narisetti *et al*., 2020; Han *et al*., 2021). *LAR* estimation requires more complex 3D reconstruction, which is currently only possible for plant species with simple architecture, such as maize (Daviet *et al*., 2022).

Depending on target variables, HTP may be able to contribute significantly to the parameterization and evaluation of PBMs (Box 1). Application of HTP for estimation of highly predictable variables summarized here can help scale the application of models (e.g. across diverse germplasm) by directly parameterizing model inputs or gathering time‐series trait estimates to be used for inverse modeling (e.g. Jin *et al*., 2018) or evaluation efforts (Fig. 4). The model variables for which HTP approaches had moderate applicability may additionally need estimation of prediction errors to characterize how uncertainty propagates in PBMs when using these indirect measures as parameters. For traits that cannot yet be measured consistently using HTP approaches, such as dark respiration or root growth traits, and whose roles in whole‐plant physiology require further elucidation, progress to advance phenotyping may simultaneously increase understanding of their physiological impacts.

## What is needed to improve integration between high‐throughput phenotyping and process‐based modeling?

Excitingly, PBMs have the potential to serve as a bridge to translate results across different experimental settings, adding mechanistic value to advancements made in indoor and outdoor HTP. However, broader implementation of integrated HTP–PBM experiments is challenged by several issues, including the need to reconcile scale differences between phenotyping and model variables and to align model representation with the research questions at hand. Below, we discuss three emerging areas in the integration of HTP and PBM:

### Use of controlled‐environment HTP data for modeling

Existing crop and vegetation PBMs generally simulate field‐level processes, giving rise to natural alignment (with respect to spatial scale) with field‐based HTP approaches for model parameterization or evaluation. Indeed, the integration of remotely sensed data and process‐based crop modeling has been an active area of research (Jin *et al*., 2018). By contrast, controlled‐environment‐based phenotyping methods typically assay at the individual plant level (and have the potential to extract organ‐level traits), making it less straightforward to leverage these data directly for parameterization/evaluation of existing models designed for field‐level simulation. Model state variables such as *LAI* (m^2^ leaf area m^−2^ land area), essential for scaling canopy‐level processes in existing PBMs, may not have a straightforward or relevant analog under controlled environments (Bouidghaghen *et al*., 2023). Use of plant trait ontologies could facilitate more interoperability between HTP products and PBM variables (Box 2).

Box 2 **Can the use of ontologies improve mapping of HTP‐derived traits to PBM variables?**

A key challenge in using high‐throughput phenotyping (HTP) to parameterize or evaluate process‐based models (PBMs) lies in identifying which phenotyping methods are most suitable for estimating specific model variables. Figure 3 and Table S1 from this review aim to provide a starting point for experimentalists for some of the most common model variables. However, as new methods and models emerge, a more systematic and scalable approach is needed to align HTP‐based trait predictions with the variables critical to PBMs.

Greater adoption of ontologies in phenotyping studies may help facilitate this. Ontologies are controlled vocabularies designed to annotate data in accordance with Findable, Accessible, Interoperable, and Reusable (FAIR) principles (Arnaud *et al*., 2020). They can help standardize trait descriptions and facilitate cross‐study comparisons, for example by labelling observed variables by their ‘trait’ (name), ‘method’, and ‘scale’ (i.e. unit; Arnaud *et al*., 2020). Several ontologies are already available to the plant science community, including Plant Trait Ontology (Cooper *et al*., 2018) and Crop Ontology (Arnaud *et al*., 2020), the latter of which is integrated into the Minimum Information About a Plant Phenotyping Experiment (MIAPPE) framework (Papoutsoglou *et al*., 2020). The MIAPPE provides a template for organizing metadata associated with experimental studies. Additionally, resources are available to search existing ontologies, such as the EMBL‐EBI Ontology Lookup Service (https://www.ebi.ac.uk/ols4/), AgroPortal (https://agroportal.lirmm.fr), and The Planteome Project (https://browser.planteome.org/amigo). These services support cross‐referencing between various ontologies developed by different communities. Despite the availability of ontology resources, they have not been widely adopted across HTP studies, which may be due to the current effort required to annotate data; new tools to automate these processes are under development (Arnaud *et al*., 2020). By being able to easily access and filter annotated trait data that result from phenotyping studies, researchers interested in leveraging HTP methods for PBM application may more efficiently identify methods that serve as promising proxies for model variables.

Despite the difference in scale between controlled‐environment‐derived plant data and variables in existing field‐based PBMs, several studies have successfully leveraged CE‐based phenotyping for modeling and vice versa. Lacube *et al*. (2020) developed a maize leaf growth model that could be parameterized using previous CE‐based HTP to record individual leaf growth. The model was able to be validated with field experiments on numerous maize hybrids and recombinant inbred lines across multiple years and locations. This study demonstrated that when well‐parameterized PBMs serve as an intermediary to account for new environmental conditions, CE results could be extended to diverse field conditions across numerous genotypes. More recently, Bouidghaghen *et al*. (2023) systematically summarized traits that could be measured using CE‐based robotized platforms and translated to field conditions by considering explicit environmental conditions, such as through a process‐based crop model. Going in the other direction, first principle‐based PBMs have been applied to add functional meaning to CE‐based HTP measurements. For example, leaf area and pot weight data collected with high‐throughput approaches have been used to invert the Penman–Monteith model for estimation of genotype‐specific whole‐plant maximum stomatal conductance (Prado *et al*., 2018; Bouidghaghen *et al*., 2023). These studies exemplify ways that controlled‐environment HTP and PBMs can complement each other to improve understanding of plant growth behavior under different environmental scenarios.

### Aligning model representation to match scale and complexity of research questions

Notably, the Lacube *et al*. study described above required model modifications to provide more detailed representation of growth to address their research questions. These changes accordingly matched what could be gathered from their CE‐based platform. We suggest, for studies aimed at simulating intraspecific genetic variation, model design can consider parameters that vary across genotypes, including environmental sensitivities. This highlights the general importance of tailoring model representation to the physiological detail required by the research question. For example, in order to simulate the ability of different crop genotypes to cope with a range of water deficit scenarios via acclimating processes (e.g. changes in stomatal conductance, tissue hydraulic conductance, and leaf/growth growth), models will need to account for these processes. As current agricultural PBMs of plant growth rely on empirical relationships between soil water reserve and transpiration or growth, this would require changes in model formalisms to explicitly simulate water transport processes (i.e. fluxes, potentials, and conductances). Efforts to represent more detailed processes underlying water stress, including plant hydraulics, are already being carried out in plant ecological PBMs (Trugman *et al*., 2019; Dukes *et al*., 2026) and state of the art models include those that represent xylem vulnerability to cavitation (Mackay *et al*., 2015). To understand which level of process abstraction may be appropriate, several tools are available for crop and vegetation models to compare different representations of physiological mechanisms (Dietze *et al*., 2013; Walker *et al*., 2021; Lochocki *et al*., 2022). These are additionally useful for quantifying uncertainty resulting from varying model parameters or representations (Fisher *et al*., 2015).

### Use of HTP to enhance trait‐based parameterization for regional to global scale models

PBMs developed for regional and global scale studies share similar physiological processes with models designed for use at organ and plant scales, but they emphasize scalability in space over representation of individual plants. They traditionally scale up to areas using either plant functional types (PFTs) arranged using a ‘big‐leaf’ analogy and subdividing a region into fractional areas, or cohorts of PFTs that compete for resources, such as light, water, and nutrients, within the same area. PFTs are usually defined in terms of leaf phenology (i.e. evergreen and deciduous), leaf morphology (i.e. needleleaf and broadleaf), growth form (e.g. tree, shrub, grass, and crop), and photosynthetic pathway. Many global scale PBMs make a distinction between plant processes at the conceptual level of organs (i.e. leaf, stem, root; Trugman *et al*., 2019), and the cohort models also retain the conceptual idea of organisms, particularly with respect to height dominance. Recently, global PBMs have been moving toward representing PFT distributions as ecological responses to climate and soil instead of prescribed based on climatic limits to their growth or historical boundaries between vegetation types (Fisher *et al*., 2015). As they have grown in sophistication so have their needs for observation based functional traits. Given their spatial scales, these models have relied primarily on HTP from space‐borne sensors, but the forefront of research is in merging HTP data with region‐ or species‐specific plant traits held in harmonized databases (Dukes *et al*., 2026). A mismatch still exists between data directly retrievable through HTP, such as the spectral properties of canopies, which are phylogenetically conserved, and the small number of PFTs used in global models to represent all land plants. For example, the hyperspectral signature of *V*_cmax_ (Table 2) at a given location may not be biologically possible for the PFT used by the model at that location. In contrast to traditionally grouped PFTs, Lineage Functional Types (LFTs) have recently been proposed (Anderegg *et al*., 2022). This framework categorizes plants according to their evolutionary lineages and is based on the concept of niche conservatism whereby closely related taxa share ecological and functional characteristics. We suggest a move toward LFTs in global models could bridge this gap between HTP and PFTs to better align model representation of functional traits using information retrieved from sensors.

## Conclusions

This review highlights the potential for systematic linkages between HTP with PBMs of plant growth (Fig. 4). An integrated approach has the potential to advance both understanding and prediction in ways beyond what either can achieve on their own, with diverse applications across the plant sciences (Box 3). When and how (e.g. in which direction?) to integrate these approaches depends on the research questions at hand as well as the study species. In particular, the use of CE‐based phenotyping for parameterizing models that can be used for field‐level prediction (Prado *et al*., 2018; Bouidghaghen *et al*., 2023) holds promise for forecasting the behavior of large panels of genotypes under new environmental scenarios. Notably, the above mentioned example of this (Lacube *et al*., 2020) was carried out in maize, a species characterized by simple plant architecture. Extending this to plants that feature tillering/branching or those that have indeterminate growth is still challenging. For example, organ occlusion and the nondiscrete nature of phenology give rise to considerable difficulty in quantifying model growth parameters through the identification of visual markers (e.g. via RGB imaging). However, there is much interest in this space (Narisetti *et al*., 2020) and future advancements in engineering and computation will likely provide solutions. From the plant biology perspective, there are fundamental gaps in knowledge of processes like hormone cross‐talk, dark respiration, source–sink balance and root costs, along with plant–microbiome interactions and the genetic bases of all these; such functions may need to be represented in models to achieve the level of predictive capacity required for translational endpoints, such as crop breeding. Addressing questions of regional and global scale in natural systems, HTP approaches can help support the move toward more biologically meaningful LFT‐based modeling. Continued collaboration between engineers who design and improve sensors, platforms, and analysis algorithms and process‐based modelers who can refactor plant development representation to reflect new applications and data will be essential to overcoming these challenges. By working with these interdisciplinary teams, plant scientists will be able to advance understanding and prediction of organismal response to the environment across diverse species and genotypes, with potential translational impacts for plant breeding and ecosystem management (Jung *et al*., 2021; Pal *et al*., 2025).

Box 3 **Advancing plant sciences through HTP–PBM integration**

Below, we provide a few specific examples of how the integration of high‐throughput phenotyping (HTP) and process‐based model (PBM) can be leveraged to move the plant sciences forward in ways that are beyond what either approach can achieve on their own.

*Plant genetics: elucidating the genetic architecture of plant hydraulic traits*. HTP of model state variables across large mapping populations can be used in an inverse‐modeling framework (Fig. 4c) to estimate genotype‐specific parameters for traits that are not feasible to assay with conventional phenotyping. Hydraulic vulnerability curve parameters (e.g. from the PBM, TREES (Mackay *et al*., 2020)), which describe embolism response to decreasing xylem pressure, are one such example. Despite evidence that their values may be specific to genotype (Wang *et al*., 2020) and have a large influence on physiological response to drought, little is known about the genetic controls on their observed intraspecific variation. HTP approaches in conjunction with inverse PBM methods would enable the scale‐out of hydraulic parameter estimation for large germplasm panels to be used for downstream genetic mapping.

*Crop physiology: improving predictions of nitrogen demand*. Plant nitrogen (N) dilution curves, which quantify the decreasing response of tissue nitrogen content with increasing tissue size, play a central role in plant PBMs to simulate N demand and uptake (Stöckle *et al*., 1994). This relationship varies with tissue type and developmental stage (Ata‐Ul‐Karim *et al*., 2017) yet is rarely parameterized with this level of detail. HTP‐based approaches, which have demonstrated strong predictive capacity for leaf nitrogen (Fig. 3), offer a promising means to efficiently parameterize these curves. These data can enhance simulation accuracies to support improved modeling of nitrogen‐dependent physiological processes, such as carbon assimilation, as well as to enable more precise crop nitrogen management.

*Plant ecology: understanding drivers of community shifts*. Integration of HTP and PBMs may also hold promise for improving understanding of landscape‐scale processes. For example, HTP methods can expand the applicability of PBMs to ecosystems that are hitherto poorly represented, such as herbaceous ecosystems. As suggested by Wilcox *et al*. (2023), there are several needs in this research space that could be addressed with this approach, namely the use of aerial imaging scanners to represent greater diversity of vegetation types for modeling community dynamics. These improved models have potential to overcome current challenges in accurately simulating observed herbaceous community shifts.

## Competing interests

None declared.

## Author contributions

DRW designed and supervised the review process. TT carried out the literature review. DRW and TT drafted the manuscript. DSM, JJ, MPR and YY contributed writing and/or editing to the manuscript.

## Disclaimer

The New Phytologist Foundation remains neutral with regard to jurisdictional claims in maps and in any institutional affiliations.

## Supporting information


**Fig. S1** Plant traits as variables of process‐based models.
**Fig. S2** Current status of high‐throughput approaches for estimating process‐based model variables along with references.
**Methods S1** Methodology for literature survey.
**Notes S1** Results of literature survey.

**Table S1** Summary of original studies that utilized high‐throughput phenotyping approaches to estimate variables in process‐based models.
Please note: Wiley is not responsible for the content or functionality of any Supporting Information supplied by the authors. Any queries (other than missing material) should be directed to the *New Phytologist* Central Office.
