# Supplementary material for: Beyond high‐throughput: leveraging plant phenotyping to improve understanding and prediction of plant growth through process‐based models
Source: New Phytol. 2026 Mar 2;250(3):1468–82. doi: 10.1111/nph.71039 (PMC13062729; doi:10.1111/nph.71039)
Supplement: Supplementary file 1 — Fig. S1 Plant traits as variables of process‐based models. Fig. S2 Current status of high‐throughput approaches for estimating process‐based model variables along with references. Methods S1 Methodology for literature survey. Notes S1 Results of literature survey. [file NPH-250-1468-s002.docx]

## *New Phytologist* Supporting Information

Article title: Beyond high-throughput: leveraging plant phenotyping to improve understanding and prediction of plant growth through process-based models

Authors: To-Chia Ting, D. Scott Mackay, Jinha Jung, Matthew P. Reynolds, Yang Yang, Diane R. Wang

Article acceptance date: 03 February 2026

The following Supporting Information is available for this article:

**Table S1.**  Summary of original studies that utilized high-throughput phenotyping approaches to estimate variables in process-based models

**Figure S1.**  Plant traits as variables of process-based models (PBMs).

**Figure S2.**  Current status of high-throughput approaches for estimating process-based model variables along with references.

**Methods S1.**  Methodology for literature survey.

**Notes S1.**  Results of literature survey.

**Figure S1.**  **Plant traits as variables of process-based models (PBMs).** Tables in **(a-c)** show input and output variables from component models that frame this review, ranging from simple to more complex representations (columns from left to right). Model reference numbers in **(a)** C1: Radiation Use Efficiency model (Monteith, 1972), C2: the C3 Farquhar, von Caemmerer and Berry biochemical model (FvCB) (Farquhar *et al.*, 1980), C3: sunlit/shade scaling for FvCB (De Pury and Farquhar, 1997) and C4: multi-layer scaling for FvCB (Goudriaan J., 1977; Norman JM, 1982); in **(b)** W1: WOFOST, W2: APSIM, W3: EPIC, W4: DSSAT, W5: SWAP and W6: CropSyst, each referenced in (Camargo and Kemanian, 2016); and in **(c)** N1: EPIC (Sharpley and Williams, 1990), N2: CropSyst (Stöckle *et al.*, 1994), N3: CERES-N (Godwin and Allan Jones, 1991) and N4: HERMES (Kersebaum, 1995, 2007). Asterisks in **(a)** indicate variables that are specific to leaf-angle class in the multi-layer canopy scaling model shown in the last column while asterisks in **(c)** indicate model variables that are specific for roots or shoots. Variable names written in bold italicized text indicate output variables while regular text denotes input variables. This figure was created in BioRender (https://BioRender.com/pv9ghey).

**
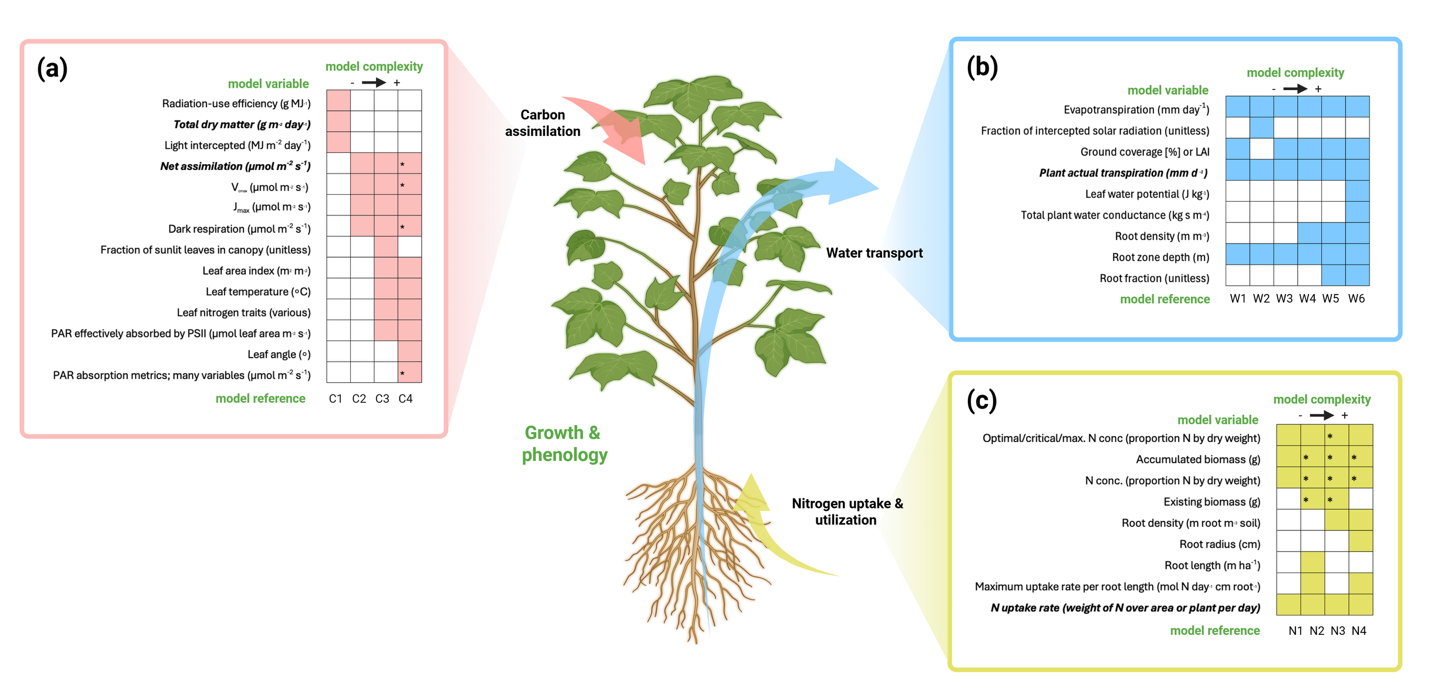
**

**Figure S2.**  **Current status of high-throughput approaches for estimating process-based model variables along with references.** This figure shows the same information as in Figure 2 of the main manuscript with the addition of supporting references added directly below each trait. This figure was created in BioRender (https://BioRender.com/vrjoqvk).

**
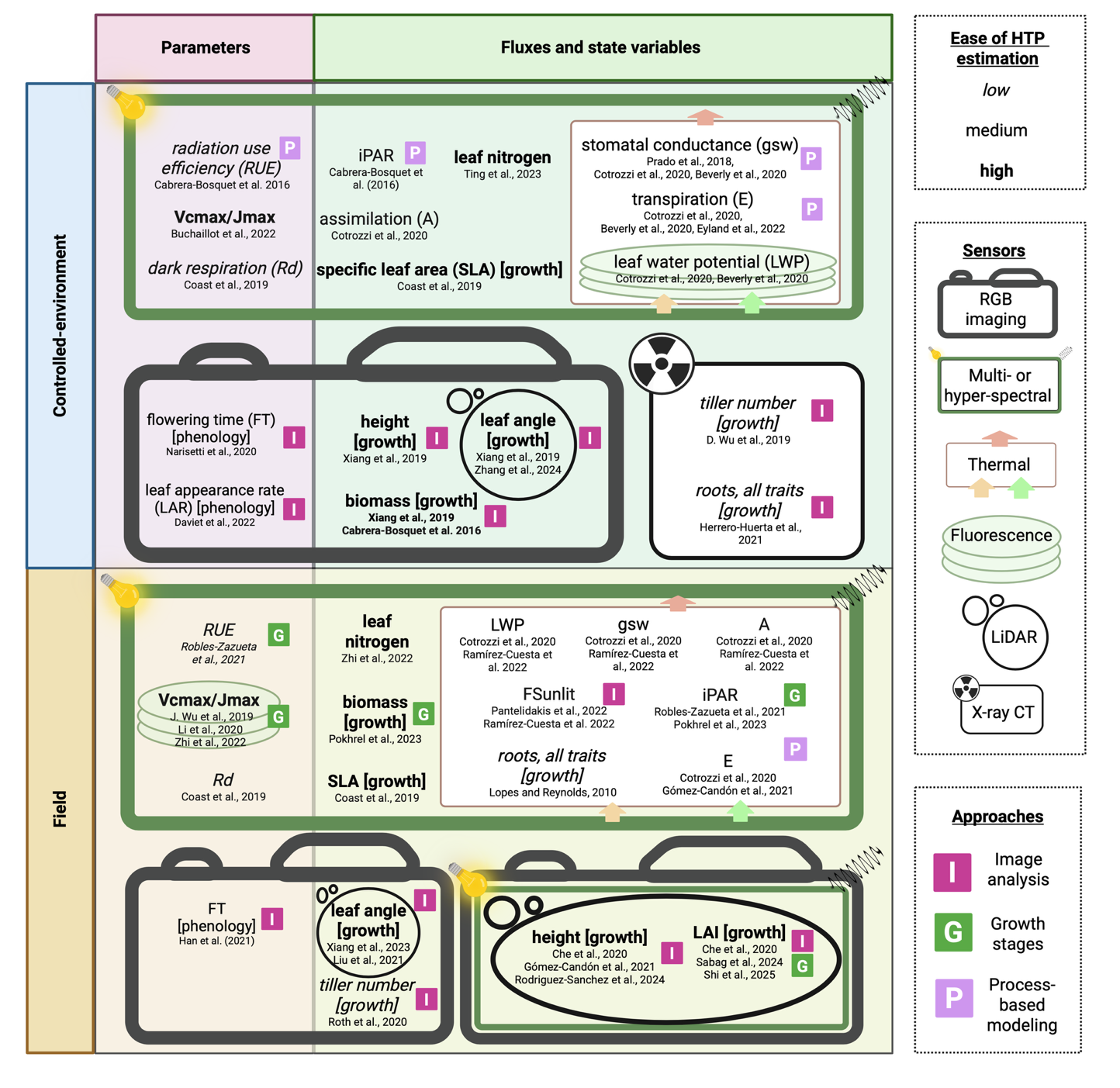
**

**Methods S1.**  Methodology for literature survey.

To evaluate the utility of high-throughput phenotyping approaches to estimate variables used in physiological process-based models, studies were gathered using the Scopus database for those that contained the terms “high-throughput phenotyping” or “phenomics” or “non-destructive” and the trait of interest in the article title, abstract, or keywords. Articles were filtered for those that were published in the time range, 2019-2024, however, earlier seminal studies on root phenotyping or studies that demonstrated unique methodologies published prior to 2019 were also considered. The final collection was selected to represent diverse species, approaches, and experimental conditions. To assess the applicability of high-throughput methods for model variable prediction, correlation coefficient was used as the primary indicator of model performance, as it was the most common metric among the studies from which regression models were developed. For studies where classification models were developed, model performance was not compared. Results with correlations above 0.8 were classified as having strong predictability, 0.5 to 0.8 indicating moderate predictability, and below 0.5 indicating weak predictability. Predictions using high-throughput approaches were additionally evaluated on their broad applicability across species, genotypes, and environmental settings. Results are summarized in **Figures 3 and S2, Table 2** and **Table S1**. **Table S1** is a subset of a larger literature review available at Zenodo (doi:  [10.5281/zenodo.15008712](https://doi.org/10.5281/zenodo.15008712)).

**Notes S1.**  Results of literature survey.

*Estimation of carbon assimilation traits:* Survey results show that hyperspectral sensing plays a key role in estimating model variables related to carbon assimilation. In the radiation use efficiency (*RUE*) model, the input variable (*RUE*, [g MJ^-1^]) could be estimated using hyperspectral reflectance with moderate accuracy under field conditions in wheat (11 varieties) (Robles-Zazueta *et al.*, 2021) and cotton (single variety) (Pokhrel *et al.*, 2023). However, prediction depends on growth stage (Robles-Zazueta *et al.*, 2021) and growth habit (e.g., determinate or indeterminate) (Pokhrel *et al.*, 2023), underscoring the fact that *RUE* is a rather high-level, integrated variable that is influenced by both environmental and biological factors; this may limit the generalizability of its prediction. The radiation use efficiency model additionally requires estimates of intercepted photosynthetically active radiation, *iPAR* [MJ m^-2^] as an input. Recent studies have suggested that *iPAR* can be predicted well from vegetation indices (VIs) and canopy temperature (Robles-Zazueta *et al.*, 2021), although again, including phenology information is essential (Pokhrel *et al.*, 2023). In addition to predicting *RUE* directly from hyperspectral data, this parameter can be estimated using a 3D growth model that leverages RGB-image based biomass measures and light environment measured with a spectrometer; while this approach is complex, researchers have used it with success to detect a seasonal pattern to *RUE* [g fresh weight PPFD mol^-1^ plant^-1^] (Cabrera-Bosquet *et al.*, 2016).

In contrast to the radiation-use efficiency model of carbon assimilation, the FvCB biochemical model of C3 photosynthesis includes input and output variables at a physiologically lower level, e.g., dark respiration (*Rd*, [$\mu$mol O_2_ m^-2^ s^-1^]), maximum rate of Rubisco catalyzed carboxylation (*Vcmax*, [$\mu$mol m^-2^ s^-1^]), maximum rate of electron transport (*Jmax*, [$\mu$mol m^-2^ s^-1^]), and net assimilation (*A*, [$\mu$mol m^-2^ s^-1^]). Recent phenotyping studies show that these traits generally had more consistent predictions using HTP approaches than *RUE*. Specifically, *A* could be estimated through hyperspectral reflectance moderately well, as in studies involving maize (Cotrozzi *et al.*, 2020) and peanut and soybean (Buchaillot *et al.*, 2022) (n = 6 genotypes each). Thermal information can also be used to estimate *A*, as in a peach tree study led by Ramírez-Cuesta et al. (2022). This may be due to the close functional relationship between stomatal conductance (*gsw*) and *A*, with *gsw* estimable from thermal data (see section below, *Estimation of water transport traits*). For *Rd*, which often requires nighttime measurements, this is not commonly addressed using HTP methods. However, a single study found moderate predictability for *Rd* in 90 wheat genotypes using hyperspectral reflectance (Coast *et al.*, 2019).

Of the C3 carbon assimilation model traits, *Vcmax* and *Jmax* are the most predictable. Studies using hyperspectral reflectance or SiF show high-moderate to high predictability for these parameters (Wu *et al.*, 2019*b*; Li *et al.*, 2020; Meacham-Hensold *et al.*, 2020; Buchaillot *et al.*, 2022; Zhi *et al.*, 2022). Again, predictions are largely growth-stage dependent (Wu *et al.*, 2019*b*; Li *et al.*, 2020). Various scaling options are available for the leaf-level FvCB photosynthesis model; these options depend on additional parameters such as the fraction of sunlit leaves (*FSunlit*, unitless), *LAI,* or *leaf angle*. To predict *FSunlit,* radiance data from hyperspectral image and thermal images are each able to classify sunlit/shade leaves (Meacham-Hensold *et al.*, 2020; Pantelidakis *et al.*, 2022; Ramírez-Cuesta *et al.*, 2022), typically through the application of machine learning algorithms. *LAI* and *leaf angle* are discussed in the section, *Estimation of phenology and growth traits.*

*Estimation of water transport traits:* Diverse sensing approaches are available for estimating water status traits, including hyperspectral, thermal, and RGB sensors. Leaf-level stomatal conductance (*gsw*, [mol m^-2^ s^-1^]) and transpiration (*E*, [mmol m^-2^ s^-1^]) can be predicted, with high-moderate to high correlations, using the visible and near-infrared (VNIR) hyperspectral region (maize - (Cotrozzi *et al.*, 2020)). Based on the principle that water flux affects plant energy balance, thermal data may also be used to estimate leaf-level *gsw*; e.g., in peach trees in field conditions (Ramírez-Cuesta *et al.*, 2022), and trees, oilseed, and sagebrush in controlled environments (Beverly *et al.*, 2020), each with high-moderate to high predictability. Thermal data may also be combined with energy balance models to estimate plot-level *E* [mm hr^-1^] (Gómez-Candón *et al.*, 2021) and leaf-level *E* [mmol m^-2^ s^-1^] (Beverly *et al.*, 2020) with moderate to strong predictability. Estimation of plant transpiration can also take advantage of RGB imaging in conjunction with load-cell systems under controlled environments. This method involves logging weight at high temporal resolution to estimate water loss between timesteps, which is then divided by leaf area modeled from RGB images. Using this method, variations in diurnal dynamics whole-plant-level *E* [g cm^-2^ h^-1^] can be computed, as was carried out for eight banana wild relatives (Eyland *et al.*, 2022), enabling the parameterization and evaluation of a biophysical model. Furthermore, whole-plant level maximum *gsw* may be estimated by inverting Penman-Monteith equation with an input of *E*, derived from the approach described above (Prado *et al.*, 2018). In addition to *gsw* and *E*, leaf water potential (*LWP* [-MPa]) is an important trait in water transport models. Estimation of *LWP* can be carried out using hyperspectral sensors (Cotrozzi *et al.*, 2020), although use of thermal and/or fluorescence data yield stronger predictions, likely due to the fact that changes in *LWP* (or stem water potential) reflect changes in energy balance (Beverly *et al.*, 2020; Ramírez-Cuesta *et al.*, 2022).

*Estimation of nitrogen traits:* Recent phenotyping studies demonstrate that leaf nitrogen (*leafN*, various units [**Table S1**]) can be proxied well with hyperspectral data across various types of crops and environmental conditions. For example, this has been shown in sorghum for over 100 genotypes under field conditions (Zhi *et al.*, 2022), 23 rice genotypes in growth chamber environment (Ting *et al.*, 2023), 90 wheat genotypes in both controlled and greenhouse conditions (Coast *et al.*, 2019), and 282 maize genotypes in both field and greenhouse settings (Ge *et al.*, 2019). This high confidence in predicting *leafN* with hyperspectral reflectance is beneficial for applying PBMs across large experiments, as it reduces the need for laborious destructive sampling typically required at multiple timepoints. However, predicting nitrogen content in organs beyond leaves remains challenging (Prey *et al.*, 2020). Additionally, since prediction relies on the functional link between nitrogen content and chlorophyll, it provides limited insight into its translocation. understanding nitrogen allocation could be improved by using spectral regions directly related to nitrogen associated with protein, such as signals in the short wave infrared region (Berger *et al.*, 2020).

*Estimation of phenology and growth traits*: Phenology affects plant structure, which in turns affects nearly all plant physiological processes. As such, there have been a variety of high-throughput means that have been tested to record the timing of plant biological events, both at the individual plant scale as well as at the regional or field levels. Under controlled environments, prediction of *flowering* time (*FT*) may be carried out using information gathered from RGB images; for example, this has been demonstrated in wheat (withing 52 genotypes) on individual plants with high correlations (Narisetti *et al.*, 2020). In outdoor settings, multispectral and NIR-green-blue sensors deployed on UAS are able to estimate *FT* with the use of machine learning algorithms for image classification. Using this approach, Han et al. (2021) found two independent quantitative trait loci that explained more than 30% of the phenotypic variation in *FT* of 240 lettuce genotypes. In contrast to multispectral and RGB sensing from UAS, hyperspectral sensing from this same platform has been less extensively tested, likely due to its comparatively complicated radiometric and geometric calibration processes. This gap represents a promising area for future research. Other measures of phenology, such as *leaf appearance rate* (*LAR*), may be monitored under controlled environments using multi-angle side-view RGB imaging in conjunction with sophisticated 3D + t (3-dimensional space plus time) reconstruction; however, this is currently only possible in plants with simple architecture, such as maize (Daviet *et al.*, 2022).

For aboveground growth traits, we evaluated a sample set representing size traits, mass traits, and indices (Hilty *et al.*, 2021). Among all growth traits, *height* (a 1-D size trait) is predicted most consistently and accurately (correlations close to 1) through the use of digital surface or digital terrain models (Che *et al.*, 2020). Indeed, this is a widely used output from automated controlled environment phenotyping facilities using standard RGB image processing pipelines (e.g., PlantCV, an open-source image analysis package (Gehan *et al.*, 2017)). As 3D reconstruction becomes more commonplace, *height* can be estimated together with other architectural traits, such as *leaf angle* in indoor conditions (Xiang *et al.*, 2019) or *LAI* in outdoor conditions (Che *et al.*, 2020). Various instruments may be used to generate 3D models, such as multi-angle nadir and/or oblique RGB imaging (e.g., maize (Che *et al.*, 2020); sesame (Sabag *et al.*, 2024)), multispectral sensor (wheat (Gómez-Candón *et al.*, 2021)) or LiDAR (cotton (Rodriguez-Sanchez *et al.*, 2024)). Another common HTP product is leaf area (a 2D size trait). While leaf area is not part of the component models outlined in the current review, it is worth mentioning that estimation of leaf area paved the way for computation of *iPAR* and *E* (Cabrera-Bosquet *et al.*, 2016; Eyland *et al.*, 2022) and a precursor to *LAI* estimation (Liu *et al.*, 2021). For estimation of *leaf angle*, LiDAR may be applied with high accuracy under outdoor (maize (Liu *et al.*, 2021)) and indoor (soybean (Zhang *et al.*, 2024)) conditions. As a model variable, *leaf angle*, in conjunction with *leaf area*, is important for scaling up photosynthesis from leaf-level to canopy-level (Liu *et al.*, 2021) and for predicting *iPAR* (Cabrera-Bosquet *et al.*, 2016).

Size traits further facilitate estimation of plant *biomass* [g plant^-1^] (a mass trait) in controlled environments (Cabrera-Bosquet *et al.*, 2016; Xiang *et al.*, 2019). In field settings, estimations of *biomass* [g m^-2^] have moderate to strong predictability using various predictors, such as the combination of growing degree days and VIs from multispectral sensors (cotton (Pokhrel *et al.*, 2023)), or VIs from hyperspectral sensor collected at leaf level (wheat (Robles-Zazueta *et al.*, 2021)). Various combinations of information can be used to estimate *biomass*, likely a reflection of the integrated influence of size, structure, and physiology on biomass.

Growth traits grouped as *indices* include ratios as well as count variables (Hilty *et al.*, 2021). *Leaf area index* (*LAI*), a critical variable in process-based models, can be predicted well across many species using various HTP approaches. Simple methods include using vegetation indices from multispectral or hyperspectral sensors (e.g., wheat (Gómez-Candón *et al.*, 2021) and sesame (Sabag *et al.*, 2024)) while more sophisticated methods may include analyzing 3D point clouds generated from RGB images (maize, (Che *et al.*, 2020)) and LiDAR (e.g., soybean (Shi *et al.*, 2025) and maize (Liu *et al.*, 2021)). Inclusion of phenology may be necessary when using only VIs (Gómez-Candón *et al.*, 2021). *LAI* derived from 3D modeling may be able to inform multi-layer photosynthesis models, which require inputs of *LAI* at individual canopy layers (**Figure S1**). In comparison to *LAI*, the estimation of *specific leaf area* (*SLA*) (or its inverse leaf mass area), has received relatively less attention, despite its importance as a functional plant trait and central role in the leaf economic spectrum (Wright *et al.*, 2004). In the studies available, *SLA* is predicted with high-moderate to high correlations with hyperspectral sensors (maize (Ge *et al.*, 2019; Cotrozzi *et al.*, 2020); sorghum (Zhi *et al.*, 2022); and wheat (Coast *et al.*, 2019)), likely due to its relationship with nitrogen traits. In addition to crop species, *SLA* is also well predicted in trees, shrubs and forbs in forests (Ali *et al.*, 2017). Finally, we reviewed one count trait, *tiller number*, because of its fundamental role in the growth of economically important grass species. Estimation of *tiller number* typically requires 3D analysis, where self-occlusion poses a major challenge. As a result, estimation is most successful in early growth stages (e.g., wheat (Roth *et al.*, 2020); rice (Wu *et al.*, 2019*a*)), leveraging various sensors including RGB imaging and X-ray CT.

Although belowground traits can also be classified using the size-mass-indices framework, we considered all *root traits* together here due to their unique challenges in sample accessibility. Under controlled environments, the HTP community has primarily tried to address these challenges with the use of (1) transparent growth media (Zhu *et al.*, 2022) or (2) technologies that enable non-destructive sensing (e.g., X-ray CT (Herrero-Huerta *et al.*, 2021)). Under field settings, canopy temperature is shown to predict *dry weight* of roots below 0.5m in wheat under gravity drought stress with high precision (Lopes and Reynolds, 2010) and differentiate root capacity at high vapor pressure deficit (Pinto and Reynolds, 2015). Root system images can also be used to estimate PBM-relevant traits such as *root length* (Herrero-Huerta *et al.*, 2021; Zhu *et al.*, 2022), *root radius* (Zhu *et al.*, 2022), and *root density* (Herrero-Huerta *et al.*, 2021; Zhu *et al.*, 2022), but sampling remains a major challenge. Advancements in (1) technologies to generate consistent data under various experimental conditions (e.g., X-ray CT imaging with different substrates) and (2) computational models to extract functional trait information from these data are essential to improve the representation of belowground processes in PBMs.

**References**

**Ali AM, Darvishzadeh R, Skidmore AK, van Duren I**. 2017. Specific leaf area estimation from leaf and canopy reflectance through optimization and validation of vegetation indices. Agricultural and Forest Meteorology **236**, 162–174.

**Berger K, Verrelst J, Féret J-B, Wang Z, Wocher M, Strathmann M, Danner M, Mauser W, Hank T**. 2020. Crop nitrogen monitoring: Recent progress and principal developments in the context of imaging spectroscopy missions. Remote sensing of environment **242**, 111758.

**Beverly DP, Guadagno CR, Ewers BE**. 2020. Biophysically Informed Imaging Acquisition of Plant Water Status. Frontiers in Forests and Global Change **3**.

**Buchaillot ML, Soba D, Shu T, Liu J, Aranjuelo I, Araus JL, Runion GB, Prior SA, Kefauver SC, Sanz-Saez A**. 2022. Estimating peanut and soybean photosynthetic traits using leaf spectral reflectance and advance regression models. Planta **255**.

**Cabrera-Bosquet L, Fournier C, Brichet N, Welcker C, Suard B, Tardieu F**. 2016. High-throughput estimation of incident light, light interception and radiation-use efficiency of thousands of plants in a phenotyping platform. New Phytologist **212**, 269–281.

**Camargo GGT, Kemanian AR**. 2016. Six crop models differ in their simulation of water uptake. Agricultural and Forest Meteorology **220**, 116–129.

**Che Y, Wang Q, Xie Z, Zhou L, Li S, Hui F, Wang X, Li B, Ma Y**. 2020. Estimation of maize plant height and leaf area index dynamics using an unmanned aerial vehicle with oblique and nadir photography. Annals of Botany **126**, 765–773.

**Coast O, Shah S, Ivakov A, *et al.*** 2019. Predicting dark respiration rates of wheat leaves from hyperspectral reflectance. Plant, Cell & Environment **42**, 2133–2150.

**Cotrozzi L, Peron R, Tuinstra MR, Mickelbart MV, Couture JJ**. 2020. Spectral Phenotyping of Physiological and Anatomical Leaf Traits Related with Maize Water Status. Plant Physiology **184**, 1363–1377.

**Daviet B, Fernandez R, Cabrera-Bosquet L, Pradal C, Fournier C**. 2022. PhenoTrack3D: an automatic high-throughput phenotyping pipeline to track maize organs over time. Plant Methods **18**, 130.

**De Pury DGG, Farquhar GD**. 1997. Simple scaling of photosynthesis from leaves to canopies without the errors of big-leaf models. Plant, Cell & Environment **20**, 537–557.

**Eyland D, Luchaire N, Cabrera-Bosquet L, Parent B, Janssens SB, Swennen R, Welcker C, Tardieu F, Carpentier SC**. 2022. High-throughput phenotyping reveals differential transpiration behaviour within the banana wild relatives highlighting diversity in drought tolerance. Plant, Cell & Environment **45**, 1647–1663.

**Farquhar GD, von Caemmerer S, Berry JA**. 1980. A biochemical model of photosynthetic CO2 assimilation in leaves of C3 species. Planta **149**, 78–90.

**Ge Y, Atefi A, Zhang H, Miao C, Ramamurthy RK, Sigmon B, Yang J, Schnable JC**. 2019. High-throughput analysis of leaf physiological and chemical traits with VIS–NIR–SWIR spectroscopy: a case study with a maize diversity panel. Plant Methods **15**, 66.

**Gehan MA, Fahlgren N, Abbasi A, *et al.*** 2017. PlantCV v2: Image analysis software for high-throughput plant phenotyping. PeerJ **5**, e4088.

**Godwin DC, Allan Jones C**. 1991. Nitrogen Dynamics in Soi-Plant Systems. Modeling Plant and Soil Systems. John Wiley & Sons, Ltd, 287–321.

**Gómez-Candón D, Bellvert J, Royo C**. 2021. Performance of the Two-Source Energy Balance (TSEB) Model as a Tool for Monitoring the Response of Durum Wheat to Drought by High-Throughput Field Phenotyping. Frontiers in Plant Science **12**.

**Goudriaan J.** 1977. *Crop micrometeorology : a simulation study*. Wageningen: Pudoc, Center for Agricultural Publishing and Documentation.

**Han R, Wong AJY, Tang Z, Truco MJ, Lavelle DO, Kozik A, Jin Y, Michelmore RW**. 2021. Drone phenotyping and machine learning enable discovery of loci regulating daily floral opening in lettuce. Journal of Experimental Botany **72**, 2979–2994.

**Herrero-Huerta M, Meline V, Iyer-Pascuzzi AS, Souza AM, Tuinstra MR, Yang Y**. 2021. 4D Structural root architecture modeling from digital twins by X-Ray Computed Tomography. Plant Methods **17**, 123.

**Hilty J, Muller B, Pantin F, Leuzinger S**. 2021. Plant growth: the What, the How, and the Why. New Phytologist **232**, 25–41.

**Kersebaum KC**. 1995. Application of a simple management model to simulate water and nitrogen dynamics. Ecological Modelling **81**, 145–156.

**Kersebaum KC**. 2007. Modelling nitrogen dynamics in soil–crop systems with HERMES. In: Kersebaum KC, Hecker J-M, Mirschel W, Wegehenkel M, eds. Modelling water and nutrient dynamics in soil–crop systems. Dordrecht: Springer Netherlands, 147–160.

**Li J, Zhang Y, Gu L, Li Z, Li J, Zhang Q, Zhang Z, Song L**. 2020. Seasonal variations in the relationship between sun-induced chlorophyll fluorescence and photosynthetic capacity from the leaf to canopy level in a rice crop. Journal of Experimental Botany **71**, 7179–7197.

**Liu F, Song Q, Zhao J, Mao L, Bu H, Hu Y, Zhu X-G**. 2021. Canopy occupation volume as an indicator of canopy photosynthetic capacity. New Phytologist **232**, 941–956.

**Lopes MS, Reynolds MP**. 2010. Partitioning of assimilates to deeper roots is associated with cooler canopies and increased yield under drought in wheat. Functional Plant Biology **37**, 147–156.

**Meacham-Hensold K, Fu P, Wu J, *et al.*** 2020. Plot-level rapid screening for photosynthetic parameters using proximal hyperspectral imaging. (T Lawson, Ed.). Journal of Experimental Botany **71**, 2312–2328.

**Monteith JL**. 1972. Solar Radiation and Productivity in Tropical Ecosystems. Journal of Applied Ecology **9**, 747–766.

**Narisetti N, Neumann K, Röder MS, Gladilin E**. 2020. Automated spike detection in diverse european wheat plants using textural features and the frangi filter in 2d greenhouse images. Frontiers in Plant Science **11**, 1–13.

**Norman JM**. 1982. Simulation of microclimates. Biometeorology in Integrated Pest Management. New York: Academic Press, 65–99.

**Pantelidakis M, Panagopoulos AA, Mykoniatis K, Ashkan S, Cherupillil Eravi R, Pamula V, Cruz Verduzco III E, Babich O, Panagopoulos OP, Chalkiadakis G**. 2022. Identifying sunlit leaves using Convolutional Neural Networks: An expert system for measuring the crop water stress index of pistachio trees. Expert Systems with Applications **209**, 118326.

**Pinto RS, Reynolds MP**. 2015. Common genetic basis for canopy temperature depression under heat and drought stress associated with optimized root distribution in bread wheat. TAG. Theoretical and Applied Genetics. Theoretische Und Angewandte Genetik **128**, 575–585.

**Pokhrel A, Virk S, Snider JL, Vellidis G, Hand LC, Sintim HY, Parkash V, Chalise DP, Lee JM, Byers C**. 2023. Estimating yield-contributing physiological parameters of cotton using UAV-based imagery. Frontiers in Plant Science **14**.

**Prado SA, Cabrera-Bosquet L, Grau A, Coupel-Ledru A, Millet EJ, Welcker C, Tardieu F**. 2018. Phenomics allows identification of genomic regions affecting maize stomatal conductance with conditional effects of water deficit and evaporative demand. Plant, Cell & Environment **41**, 314–326.

**Prey L, Hu Y, Schmidhalter U**. 2020. High-Throughput Field Phenotyping Traits of Grain Yield Formation and Nitrogen Use Efficiency: Optimizing the Selection of Vegetation Indices and Growth Stages. Frontiers in Plant Science **10**.

**Ramírez-Cuesta JM, Ortuño MF, Gonzalez-Dugo V, Zarco-Tejada PJ, Parra M, Rubio-Asensio JS, Intrigliolo DS**. 2022. Assessment of peach trees water status and leaf gas exchange using on-the-ground versus airborne-based thermal imagery. Agricultural Water Management **267**, 107628.

**Robles-Zazueta CA, Molero G, Pinto F, Foulkes MJ, Reynolds MP, Murchie EH**. 2021. Field-based remote sensing models predict radiation use efficiency in wheat. Journal of Experimental Botany **72**, 3756–3773.

**Rodriguez-Sanchez J, Snider JL, Johnsen K, Li C**. 2024. Cotton morphological traits tracking through spatiotemporal registration of terrestrial laser scanning time-series data. Frontiers in Plant Science **15**.

**Roth L, Camenzind M, Aasen H, Kronenberg L, Barendregt C, Camp K-H, Walter A, Kirchgessner N, Hund A**. 2020. Repeated multiview imaging for estimating seedling tiller counts of wheat genotypes using drones. Plant Phenomics **2020**.

**Sabag I, Bi Y, Sahoo MM, Herrmann I, Morota G, Peleg Z**. 2024. Leveraging genomics and temporal high-throughput phenotyping to enhance association mapping and yield prediction in sesame. The Plant Genome **n/a**, e20481.

**Sharpley AN, Williams JR (Eds)**. 1990. EPIC-erosion/productivity impact calculator: 1. Model documentation.

**Shi B, Guo L, Yu L**. 2025. Accurate LAI estimation of soybean plants in the field using deep learning and clustering algorithms. Frontiers in Plant Science **15**.

**Stöckle CO, Martin SA, Campbell GS**. 1994. CropSyst, a cropping systems simulation model: Water/nitrogen budgets and crop yield. Agricultural Systems **46**, 335–359.

**Ting T-C, Souza ACM, Imel RK, Guadagno CR, Hoagland C, Yang Y, Wang DR**. 2023. Quantifying physiological trait variation with automated hyperspectral imaging in rice. Frontiers in Plant Science **14**, 1229161.

**Wright IJ, Reich PB, Westoby M, *et al.*** 2004. The worldwide leaf economics spectrum. Nature **428**, 821–827.

**Wu D, Guo Z, Ye J, *et al.*** 2019*a*. Combining high-throughput micro-CT-RGB phenotyping and genome-wide association study to dissect the genetic architecture of tiller growth in rice. Journal of Experimental Botany **70**, 545–561.

**Wu J, Rogers A, Albert LP, Ely K, Prohaska N, Wolfe BT, Oliveira Jr RC, Saleska SR, Serbin SP**. 2019*b*. Leaf reflectance spectroscopy captures variation in carboxylation capacity across species, canopy environment and leaf age in lowland moist tropical forests. New Phytologist **224**, 663–674.

**Xiang L, Bao Y, Tang L, Ortiz D, Salas-Fernandez MG**. 2019. Automated morphological traits extraction for sorghum plants via 3D point cloud data analysis. Computers and Electronics in Agriculture **162**, 951–961.

**Zhang S, Song Y, Ou R, *et al.*** 2024. SCAG: A Stratified, Clustered, and Growing-Based Algorithm for Soybean Branch Angle Extraction and Ideal Plant Architecture Evaluation. Plant Phenomics **6**, 0190.

**Zhi X, Massey-Reed SR, Wu A, *et al.*** 2022. Estimating Photosynthetic Attributes from High-Throughput Canopy Hyperspectral Sensing in Sorghum. Plant Phenomics **2022**.

**Zhu L, Liu L, Sun H, *et al.*** 2022. The responses of lateral roots and root hairs to nitrogen stress in cotton based on daily root measurements. Journal of Agronomy and Crop Science **208**, 89–105.
